# Treatment strategy in chronic lymphocytic leukemia with symptomatic central nervous system involvement: A case report

**DOI:** 10.1002/ccr3.7965

**Published:** 2023-11-09

**Authors:** Rosina Dewaide, Kirsten Saevels

**Affiliations:** ^1^ University Hospital Antwerp, Universitair Ziekenhuis Antwerpen Antwerpen Belgium

**Keywords:** central nervous system involvement, chronic lymphocytic leukemia, treatment, venetoclax

## Abstract

**Key Clinical Message:**

This case report offers support for treatment approaches in a historically rare and very difficult to treat CLL patient population with no established guidelines.

**Abstract:**

Central nervous system involvement of chronic lymphocytic leukemia is a rare condition. Its diagnosis is often challenging, and treatment can be difficult with a lack of established guidelines. We describe a case of a 76‐year‐old male Caucasian with known chronic lymphocytic leukemia for more than 25 years, initially treated with chlorambucil. Upon first clinical relapse, cytogenetic analysis and fluorescence in situ hybridization combined showed three different abnormalities (complex karyotype), suggesting a poor prognosis. He was started on ibrutinib but developed an out‐of‐hospital cardiac arrest due to ventricular fibrillation 2 months later. Ibrutinib treatment was consequently discontinued. Due to the seriousness of the adverse event and the lack of apparent treatment indication after rapid improvement on ibrutinib, a watch‐and‐wait approach was maintained. Four years later, he developed progressive cognitive impairment, a balance disorder, and a peripheral facial nerve palsy. Anamnesis further revealed significant progressive weight loss. Routine blood tests did not show any abnormalities, but brain magnetic resonance imaging showed focal staining of cranial nerves and leptomeningeal staining. Cerebrospinal fluid analysis revealed the same monoclonal B‐cell lymphocytosis as that was already known to be present in the peripheral blood. Further analysis ruled out sample contamination or other conditions. PET‐CT scan revealed an increased uptake in the liver, and biopsy confirmed infiltration of chronic lymphocytic leukemia at the site. The patient was first started on treatment with intrathecal administration of dexamethasone, cytarabine, and methotrexate. This did not result in complete clearance in the cerebrospinal fluid. Next, oral venetoclax was initiated, resulting in rapid clearance and clinical resolution. Venetoclax, administered orally, was able to achieve clearance of the monoclonal B‐cell lymphocytosis from the cerebrospinal fluid as well as clinical response of neurological symptoms. Response was durable with persistent remission at 1 year of treatment.

## Graphical abstract

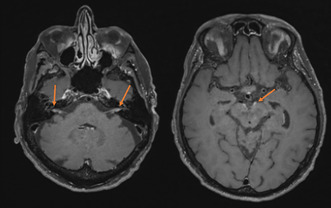

## 1 INTRODUCTION

Chronic lymphocytic leukemia (CLL) is a rather common disease in daily hematology practice, with an incidence in Belgium in 2018 of 6,1–9,3 (female–male) new cases for every 100,000 person years.^1^ Symptomatic central nervous system (CNS) involvement in patients with CLL on the other hand is very rare. However, according to autopsy studies, CNS localization may be underestimated, with some reports showing a prevalence up to 71%.^2^ Diagnosis is often a strenuous process because of the heterogenic clinical presentation. Moreover, upon finding a monoclonal B‐cell population in the cerebrospinal fluid (CSF), it can be challenging to differentiate between contamination by peripheral blood, transient presence of leukemic cells due to increased permeability of the blood–brain barrier (BBB) caused by other conditions such as inflammatory/infectious disease and clinically significant CNS involvement by CLL. The specificity of flow cytometry analysis on CSF for distinguishing symptomatic CLL involvement from other conditions is limited to 42%.^3^ Even when diagnosis of CNS involvement by CLL is made, there are no established guidelines on how to treat this entity.

## 2 CASE PRESENTATION

We present a case of a 76‐year‐old man with known chronic lymphocytic leukemia since 1993. He received chlorambucil at the time of diagnosis after which he stayed in a durable remission, occasionally needing intravenous immunoglobulins because of secondary hypogammaglobulinemia with respiratory infections. In 2005, a bone marrow examination was performed, revealing a complex fluorescence in situ hybridization (FISH) pattern with a trisomy 12 in 14/60 interphase nuclei, a deletion of 17p13.1 in 5/100 interphase nuclei and a deletion of 13q14, or monosomy 13, in 14 and 3/60 interphase nuclei. In 2017, he developed a rapidly progressive, symptomatic lymphadenopathy in his left groin. Peripheral blood counts stayed normal without lymphocytosis or cytopenia, but flow cytometry revealed a monoclonal B‐cell lymphocytosis compatible with CLL (Catovsky ≥ 4). Biopsy revealed an invasion of CLL with similar immunophenotype as peripheral blood without signs of transformation. Cytogenetics revealed trisomies 12 and 19, and FISH analysis showed a deletion of 13q14. The presence of three cytogenetic abnormalities (complex karyotype) is associated with poor prognosis.^4^ Molecular analysis was not performed. He was started on treatment with ibrutinib 420 mg per day with very rapid resolution of the palpable lymphadenopathy. Unfortunately, the patient was admitted 2 months later with an out‐of‐hospital cardiac arrest due to ventricular fibrillation, likely related to ibrutinib. Treatment was discontinued. Bone marrow examination still showed CLL invasion by 30%, cytogenetics showed the same trisomy 12 and 19, and FISH analysis showed the deletion 13q14, indicating the absence of complete response. However, due to the severity of the adverse event and no apparent clinical treatment indication after ibrutinib discontinuation, a watch‐and‐wait approach was maintained.

**FIGURE 1:**
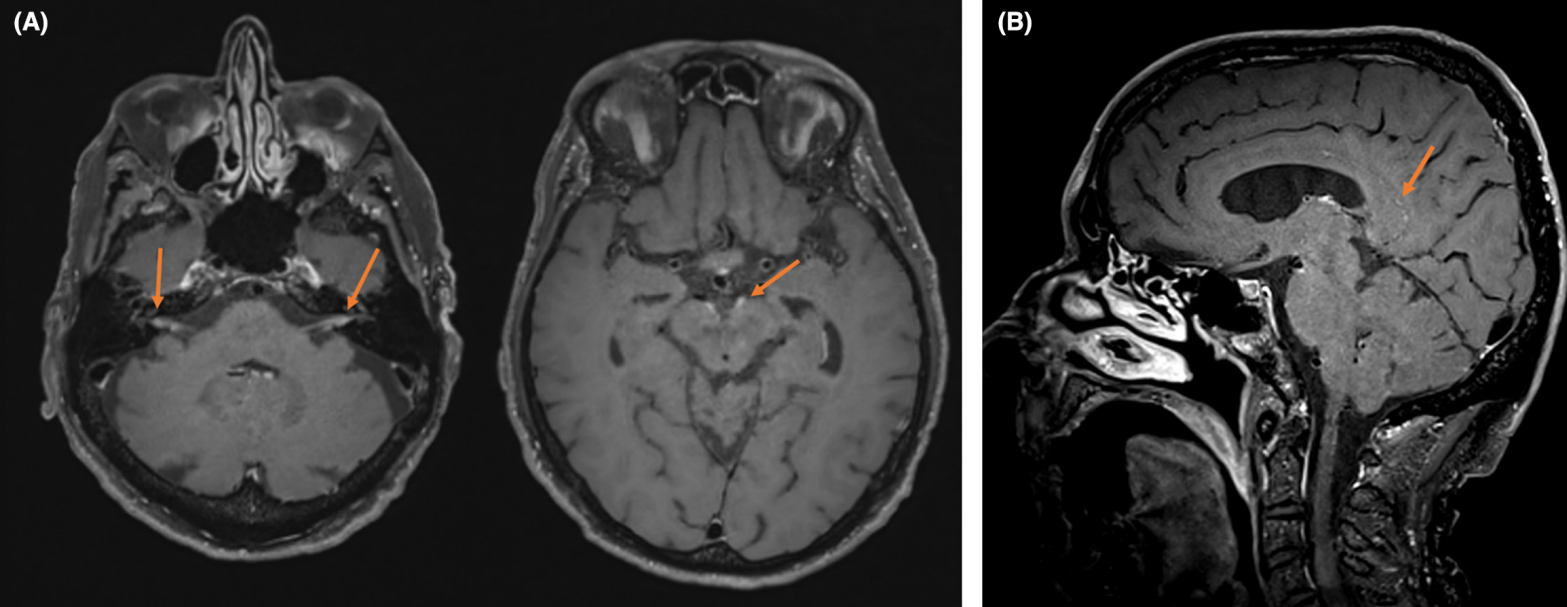
(A) Brain MRI, transverse plane. Staining of multiple cranial nerves (arrow). (B) Brain MRI, sagittal plane. Focal leptomeningeal staining in the right sulcus callosus (arrow).

In 2021, at the age of 76, the patient presented at the emergency department with progressive cognitive impairment as well as a balance disorder and a peripheral facial nerve palsy of unclear onset and was hospitalized on the neurology ward for further investigations. Anamnesis revealed no recurrent fever or night sweats, but there was unintentional weight loss of 17 kg over the course of one and a half years (>10% of his normal body weight over a period of 6 months). There were no apparent palpable lymphadenopathies or splenomegaly on clinical examination. Magnetic resonance imaging of the brain showed staining of multiple cranial nerves with focal leptomeningeal staining in the right sulcus callosus, suspect of leptomeningeal lymphoma deposits (Figure 1). Blood test again did not reveal progression of lymphocytosis or development of cytopenia. Cerebrospinal fluid (CSF) showed a high white cell count of 40/μl without erythrocytes (<1000/μl), excluding contamination by peripheral blood. There was an increased protein level and decreased glucose level. Flow cytometry on the CSF revealed a monoclonal B‐cell population with CLL phenotype. An infectious etiology was ruled out by negative viral PCR's for varicella zoster, herpes simplex and enterovirus as well as negative PCR's for Listeria and negative cultures. PET‐CT showed no evidence of FDG‐avid lymph nodes but did show diffuse heterogenic uptake in the left liver lobe. Liver biopsy surprisingly showed infiltration of CLL without signs of Richter's transformation. The diagnosis of CNS involvement of CLL together with systemic disease (liver localization) was made.

Because of its ability to penetrate through the BBB and its effectiveness in high risk (del17p) CLL, a bruton kinase inhibitor would be the preferred treatment option for this 76‐year‐old patient with a CLL with poor cytogenetics with established CNS involvement.^5^ Unfortunately, the previous history of ventricular fibrillation leading to an out of hospital arrest was regarded as a strict contraindication for ibrutinib.

We report on a different treatment strategy used in this case consisting of venetoclax in combination with intrathecal dexamethasone (4 mg), methotrexate (15 mg), and cytarabine (40 mg). The patient was first started on intrathecal (IT) chemotherapy alone on a weekly basis because of his frailty profile. A total of six IT regimens were administered. Minimal residual disease was still detected by flow cytometry on the CSF before initiating the venetoclax ramp up to 400 mg once daily. After 1 month of (ramp‐up) venetoclax, the CSF was cleared. Because of a low‐symptomatic COVID‐19 infection, rituximab was not associated as per the initial plan. Over the next few months, the patient improved considerably with clear improvement of the neurologic symptoms and Montreal Cognitive Assessment, and he had a weight gain of 10 kg. Overall, the treatment regimen was very well tolerated. Repeated flow cytometry on the CSF showed a durable response almost 1 year after start of treatment. Follow‐up PET‐CT after 1 year showed no more aberrant uptake in the liver or anywhere else.

## 3 DISCUSSION

Our rationale for choosing the BCL‐2 inhibitor venetoclax as treatment strategy for our patient was based on a few reports in the literature. There is circumstantial evidence from studies in other hematologic malignancies that oral venetoclax administration can lead to penetrance through the BBB. Venetoclax is briefly discussed in Blood (2018) as an interesting agent for primary central nervous system lymphoma (PCNSL).^6^ Also, in Blood in 2020, the passage of venetoclax into the CNS was characterized in 33 pediatric patients with relapse/refractory acute leukemia, both myeloid and lymphoid. Because venetoclax is a substrate of these efflux transporters in the BBB, one would assume there is more efflux than influx of venetoclax into the CSF. But when measuring venetoclax deposition in the CSF, the authors saw that there was still a significant concentration of venetoclax in the CSF present. So other factors/transporters must play a role. The investigators showed the ability of venetoclax to pass the BBB by measuring venetoclax concentration in the CSF (with a broad range of <0.1–13 ng/mL). However, they observed a lower‐than‐expected deposition in humans compared to mice studies where there is a higher expression of P‐gp. This suggests that other factors are involved in venetoclax disposition to the CSF.^7^ In a case report from 2019, a patient was treated with the combination of venetoclax and intrathecal cytarabine 70 mg plus methotrexate 15 mg and showed a sustained response. The authors observed that venetoclax crossed the BBB with a concentration close to half of the maximal inhibitory concentration (IC50) established in vitro in a cultured CLL cell line exposed to venetoclax for 24 h. The authors suggest that venetoclax taken orally can effectively inhibit tumor growth at the CNS site.^8^

Few other case reports mention the use of venetoclax in CNS involvement of CLL. One discusses a patient with CNS involvement of CLL treated with ibrutinib who experienced a relapse of neurological symptoms and the reappearance of a monoclonal B‐cell population in the CSF after 4 years. Venetoclax was added to ibrutinib, and this resulted in an ongoing, complete response.^9^ In another report, a patient was treated with ibrutinib 560 mg per day but was unable to achieve CSF clearance and, after 6 months of ibrutinib treatment, also had a symptomatic relapse with returning headaches and development of cervical adenopathy. Venetoclax was added, and within 2 months, the CSF was cleared. Due to reemerging headaches, thought of by the treating physician as mitigated by venetoclax, the bcl‐2 inhibitor was discontinued after 2 months, and the patient stayed on ibrutinib monotherapy. More than 1 year after the initiation of venetoclax, the CSF remains cleared.^10^

Currently, there are also two clinical trials running using venetoclax in combination with other treatment modalities in CNS lymphoma. The first trial evaluates venetoclax plus obinutuzumab for relapsed/refractory PCNSL (VENOBI‐CNS). This is a phase IB study to assess the pharmacokinetics in the CSF.^11^ The second trial aims to determine the safety and tolerability of venetoclax, ibrutinib, prednisone, obinutuzumab, and revlimid with nivolumab (VIPOR‐Nivo) in participants with PCNSL or an aggressive B‐cell lymphoma with secondary involvement of the CNS.^12^

## 4 CONCLUSION

In this case report, the clinical response as well as the durable response in CSF clearance attribute to the potential importance of venetoclax in patients with CLL with associated symptomatic CNS involvement. It offers support for treatment approaches in this historically rare and very difficult‐to‐treat CLL patient population with no established guidelines. Further research is necessary in order to determine the role of venetoclax in CNS involvement by CLL more precisely. Since adequately powered interventional studies in this rare disease setting are difficult to achieve, observational studies, either prospective or retrospective, offer more approachable research potential. A multicenter prospective observational research trial on CLL patients with established CNS involvement receiving a venetoclax‐based regimen seems to be a feasible study design and is likely to give more answers.

## AUTHOR CONTRIBUTIONS

**Rosina Dewaide:** Conceptualization; investigation; writing – original draft; writing – review and editing. **Kirsten Saevels:** Supervision.

## FUNDING INFORMATION

The author(s) received no financial support for the research, authorship, and/or publication of this article.

## CONFLICT OF INTEREST STATEMENT

The author and co‐author state that they have no conflict of interest.

## CONSENT

Written informed consent was obtained from the patient to publish this report in accordance with the journal's patient consent policy.

## Data Availability

The data that support the findings of this study are openly available.
